# Development and Validation of an Interpretable Machine Learning Model for Early Prognosis Prediction in ICU Patients with Malignant Tumors and Hyperkalemia

**DOI:** 10.1097/MD.0000000000038747

**Published:** 2024-07-26

**Authors:** Zhi-Jun Bu, Nan Jiang, Ke-Cheng Li, Zhi-Lin Lu, Nan Zhang, Shao-Shuai Yan, Zhi-Lin Chen, Yu-Han Hao, Yu-Huan Zhang, Run-Bing Xu, Han-Wei Chi, Zu-Yi Chen, Jian-Ping Liu, Dan Wang, Feng Xu, Zhao-Lan Liu

**Affiliations:** a Centre for Evidence-Based Chinese Medicine, Beijing University of Chinese Medicine, Beijing, China; b School of Traditional Chinese Medicine, Beijing University of Chinese Medicine, Beijing, China; c The Third Affiliated Hospital, Beijing University of Chinese Medicine, Beijing, China; d Department of Andrology, Dongzhimen Hospital, Beijing University of Chinese Medicine, Beijing, China; e First Clinical College, Hubei University of Chinese Medicine, Wuhan, China; f School of International Studies, University of International Business and Economics, Beijing, China; g Department of Thyropathy, Dongzhimen Hospital, Beijing University of Chinese Medicine, Beijing, China; h School of Acupuncture and Orthopedics, Hubei University of Chinese Medicine, Wuhan, China; i Department of Hematology and Oncology, Dongzhimen Hospital, Beijing University of Chinese Medicine, Beijing, China; j Surgery of Thyroid Gland and Breast, Hubei Provincial Hospital of Traditional Chinese Medicine, Wuhan, China; k Hubei Shizhen Laboratory, Wuhan, China.

**Keywords:** hyperkalemia, local interpretable model-agnostic explanations, machine learning, malignant tumors, predictive model, SHapley Additive exPlanations

## Abstract

This study aims to develop and validate a machine learning (ML) predictive model for assessing mortality in patients with malignant tumors and hyperkalemia (MTH). We extracted data on patients with MTH from the Medical Information Mart for Intensive Care-IV, version 2.2 (MIMIC-IV v2.2) database. The dataset was split into a training set (75%) and a validation set (25%). We used the Least Absolute Shrinkage and Selection Operator (LASSO) regression to identify potential predictors, which included clinical laboratory indicators and vital signs. Pearson correlation analysis tested the correlation between predictors. In-hospital death was the prediction target. The Area Under the Curve (AUC) and accuracy of the training and validation sets of 7 ML algorithms were compared, and the optimal 1 was selected to develop the model. The calibration curve was used to evaluate the prediction accuracy of the model further. SHapley Additive exPlanations (SHAP) and Local Interpretable Model-agnostic Explanations (LIME) enhanced model interpretability. 496 patients with MTH in the Intensive Care Unit (ICU) were included. After screening, 17 clinical features were included in the construction of the ML model, and the Pearson correlation coefficient was <0.8, indicating that the correlation between the clinical features was small. eXtreme Gradient Boosting (XGBoost) outperformed other algorithms, achieving perfect scores in the training set (accuracy: 1.000, AUC: 1.000) and high scores in the validation set (accuracy: 0.734, AUC: 0.733). The calibration curves indicated good predictive calibration of the model. SHAP analysis identified the top 8 predictive factors: urine output, mean heart rate, maximum urea nitrogen, minimum oxygen saturation, minimum mean blood pressure, maximum total bilirubin, mean respiratory rate, and minimum pH. In addition, SHAP and LIME performed in-depth individual case analyses. This study demonstrates the effectiveness of ML methods in predicting mortality risk in ICU patients with MTH. It highlights the importance of predictors like urine output and mean heart rate. SHAP and LIME significantly enhanced the model’s interpretability.

## 1. Introduction

Malignant tumors are complex diseases with multifactorial etiologies.^[1]^ They are among the most lethal diseases globally because of their covertness, aggressiveness, and the absence of reliable diagnostic markers. It is estimated that malignant tumors claim nearly 10 million lives annually, which is projected to rise in the future, exacerbating the strain on healthcare.^[2]^ Hyperkalemia, a common electrolyte disturbance particularly prevalent in individuals with renal dysfunction, heart failure, or those on renin–angiotensin–aldosterone system inhibitors, significantly escalates the risk of life-threatening complications, such as arrhythmias, peripheral neuropathy, and renal tubular acidosis.^[3,4]^ Hyperkalemia is also prevalent in oncology patients, often due to chemotherapy-induced tumor lysis syndrome, which causes an influx of cellular contents into the bloodstream, disrupting electrolyte balance, notably potassium levels.^[5]^ Moreover, malignant tumors and hyperkalemia (MTH) may also be closely connected with other electrolyte disturbances like hypercalcemia and hyponatremia, which are symptomatic of paraneoplastic syndromes in cancers such as breast, kidney, and prostate. Such electrolyte disorders can severely impact patient outcomes.^[6]^ Hyperkalemia has been identified as an independent predictor of increased mortality in Intensive Care Unit (ICU) patients,^[7,8]^ a finding consistent with those in Cardiac Intensive Care Unit (CICU) cohorts.^[9]^ It may add to the risks of death in ICU patients with tumors, whose death risks are high enough. The presence of malignant tumors compounds the risk of death in ICU patients, with hyperkalemia only raising the risks.

Machine learning (ML) reforms death risk prediction models, with ML-based models outperforming traditional logistic regression (LR) in handling multidimensional data and predictive accuracy.^[10]^ Kanda et al^[11]^ developed an ML model to predict adverse events in 24,949 hyperkalemic patients and validated it externally. Nonetheless, ML is a black box, thus opaque and not interpretable. Its clinical adoption is impeded.^[12]^ Therefore, it is important to elucidate the “black box” mechanism of ML by introducing the Shapley additive explanations (SHAP)^[13]^ and the Local Interpretable Model-Agnostic Explanations (LIME) techniques.^[14]^ This explains the impact of predictors on overall and individual outcomes^[15–17]^ and clarifies the probability of particular outcomes coinciding with overall outcome events. ML-based models are gaining traction across various medical domains, such as critical care,^[18]^ oncology,^[19]^ and electrolyte disorders.^[20]^

The primary objective of this study is to develop and validate a ML-based model for predicting the mortality risk of ICU patients with MTH. By analyzing clinical data from the MIMIC-IV v2.2 database, we aim to identify critical medical indicators that affect the mortality risk in MTH patients and enhance the accuracy and interpretability of predictions through ML techniques. Ultimately, this study seeks to provide clinical decision support to physicians.

## 2. Methods

### 2.1. Study design

This study developed a mortality risk prediction model for ICU patients afflicted with MTH. Seven distinct ML algorithms were employed to train the models. Subsequent validation of the model determines the most effective algorithms. Additionally, the interpretability of the model was enhanced through the application of SHAP and LIME methods.

### 2.2. Data sources and ethical review

Data for this study were sourced from the MIMIC-IV v2.2, an extensively used database developed and maintained by MIT Laboratory of Computational Physiology, and sourced from the electronic health record of the Beth Israel Deaconess Medical Center. This database includes anonymized information on demographics, vital statistics, laboratory results, and diagnostic codes, ensuring no individual patient can be identified from the data.^[21]^ Since the data for this study come from a public database, it does not involve an ethical review.

### 2.3. Sample size estimation

This study employed the 10EPV (Events Per Variable) empirical rule to determine the requisite training set sample size, ensuring robust and reliable statistical modeling. The 10EPV stipulated a minimum of 10 outcome events for each predictor variable to mitigate overfitting risks and guarantee stable parameter estimations. This approach sought to strike an optimal balance between model complexity and sample size, facilitating adequate statistical power and dependable outcomes. In this study, the initial training set included a total of 372 ICU patients with MTH.

### 2.4. Inclusion and exclusion criteria

Inclusion Criteria: ICU patients with a clinically confirmed malignancy. Patients exhibiting a peak serum potassium concentration exceeding 5.5 mmol/L.^[22]^ Exclusion Criteria: Patients with multiple ICU admissions, for whom only initial admission data were considered. Patients with an ICU stay shorter than 24 hours. Patients under the age of 18 years. In this analysis, hospital mortality among ICU patients suffering from both MTH served as the primary outcome measure.

### 2.5. Variable selection

In this study, the probability of in-hospital death in patients with MTH was a predictive outcome. The potential predictors incorporated 45 clinical laboratory measures and vital signs in the database, such as average heart rate, average respiratory rate, urine output, and peak blood urea nitrogen levels. Missing data were filled in using the multiple imputation by chained equations strategy.^[23]^ The Least Absolute Shrinkage and Selection Operator (LASSO) regression screened the selection of predictive variables,^[24]^ excluding variables with exhibiting coefficients less than zero. Additionally, to rigorously assess inter-variable relationships, a heatmap was created using the Seaborn library in Python, backed by statistical analysis via Pearson correlation coefficient.^[25]^ Variables whose correlation coefficient was 0.8 or higher were omitted to address multicollinearity.

### 2.6. Statistical analysis

We descriptively statistically analyzed the baseline characteristics of ICU patients with MTH. The cohort was stratified into 2 groups: those who experienced mortality and those who did not (non-mortality group). Following the normality test on continuous variables, we conducted an independent samples *t*-test for those adhering to a normal distribution and the Mann–Whitney *U* test for variables deviating from a normal distribution. Categorical variables were compared using the Chi square test or Fisher exact test where appropriate. The study used median and quartiles for statistical description to compare continuous variables and frequencies and percentages to compare component variables. All statistical analyses were executed using Python (version 3.6.6) and R (version 3.6.1, R Foundation for Statistical Computing), with a threshold for statistical significance set at *P* < .05.

### 2.7. Model development

In this study, in order to predict the risk of death of patients with MTH, 7 models were developed based on ML algorithms, including eXtreme Gradient Boosting (XGBoost), Random Forest (RF), Support Vector Machine, K-Nearest Neighbors (KNN), Decision Tree (DT), Naive Bayes, and Logistic Regression (LR). ICU patients with MTH were randomly divided, 75% into the training set and the remaining 25% into the testing set. The performance of the model was compared in both sets according to their accuracy and area under the receiver operating characteristic curve (ROC).^[26]^ The best prediction ML algorithm was identified and further evaluated with calibration curves to illuminate their predictive accuracy. SHAP elucidated the significance of features on individual data points, in a model’s predictions by assigning an explanatory value to each feature. SHAP summary plots illustrate the contribution of individual features to the predicted outcomes. From these summary plots, SHAP dependence plots for the top 8 influential features were generated, providing deeper insight into how these features affect the model’s predictions. SHAP force plots detail how key features impact the predictive results for individual patients.^[27]^ Additionally, the LIME method was employed to further interpret the predictions at the individual patient level.^[28]^

## 3. Results

### 3.1. Participants

**Figure 1.**
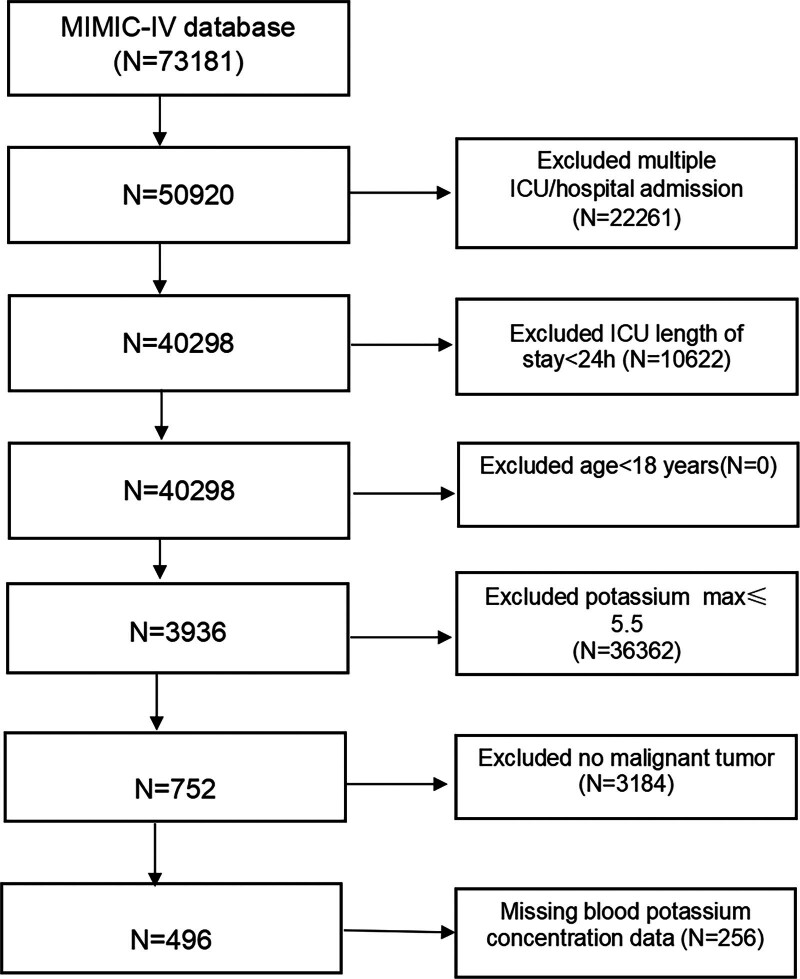
Flowchart illustrating inclusion and exclusion criteria for study cohort. MIMIC-IV, Medical Information Mart for Intensive Care.

Among the 73,181 critically ill patients sourced from the MIMIC-IV database, 22,261 patients with prior ICU admissions, 10,622 patients with ICU stays of less than 24 hours, 36,362 patients whose maximum blood potassium levels being 5.5 mmol/L or lower, 3184 not having malignant tumors, 256 patients with incomplete hyperkalemia data, are excluded. Consequently, the final study population comprised 496 MTH patients. Figure 1 illustrates the inclusion and exclusion flow chart of the study population.

### 3.2. Variants and sample size

We selected 45 clinical variables from the database as potential predictors of 24-hour in-ICU-mortality, encompassing demographic information (age, sex, weight, height, and body mass index); vital statistics (mean heart rate, blood pressure, temperature, respiratory rate, and oxygen saturation); laboratory findings (peak glucose levels, hemoglobin, white blood cell count, platelet count, bicarbonate, blood urea nitrogen, creatinine, electrolytes, prothrombin time, international normalized ratio, liver enzymes, bilirubin, calcium, cardiac and skeletal muscle enzymes, lactate dehydrogenase, serum potassium, anion gap, blood gases, hematologic indices, albumin, and urine output); and administered treatments (mechanical ventilation, sedation, and vasopressor administration). Basic baseline characteristics of 496 ICU patients with MTH are summarized in Table 1. Upon comparing baseline data, we found that among 496 patients with MTH, the median age for in-hospital survivors was 68 years old, while for those who did not survive, the median age was 71 years old. Regarding gender distribution among the survivors, there were 237 males and 113 females; in contrast, among the non-survivors, there were 97 males and 49 females. The LASSO regression analysis identified seventeen significant predictors (see Supplementary Material S1, http://links.lww.com/MD/N88), and their intercorrelations were further analyzed using heat maps. The results indicated that the correlations between these seventeen significant predictors were all below 0.8 (refer to Supplementary Material S2, http://links.lww.com/MD/N89). The training set of this investigation included 372 MTH patients, of whom 109 succumbed, yielding a mortality rate of 29.3%. Based on the 10EPV rule, our predictor variables’ calculated optimal training set sample size would be approximately 580 cases [(17 × 10) ÷ 0.293 = 580]. This calculation indicates a shortfall in our current sample size, highlighting the need for larger sample size in future studies to enhance the robustness and reliability of the statistical models.

**Table 1:** Baseline characteristics of the 496 patients with malignant tumors and hyperkalemia.

Characteristics	Non-mortality group (n = 350)	Mortality group (n = 146)	*P* value
Demographic			
Age	68.4 (61.5–78.5)	70.6 (63.4–79.6)	0.262
Gender			
Male, n (%)	237 (67.7%)	97 (66.4%)	0.864
Female, n (%)	113 (32.3%)	49 (34.6%)	
Weight, kg	80 (67.6–94.7)	74.8 (64–91.6)	0.019
Height, cm	170 (163–178)	166.5 (157–175)	<0.001
Body mass index	27.6 (23.1–32.2)	26.6 (22.9–31.3)	0.426
Vital signs			
Heart rate mean, bpm	85.5 (74.8–97.9)	97.1 (84–107.6)	<0.001
Mean systolic blood pressure, mm Hg	114.8 (105.4–124.7)	105.7 (98.4–116.9)	<0.001
Mean diastolic blood pressure, mm Hg	61 (55.1–68.6)	59.6 (52.8–64.3)	0.036
Mean blood pressure min, mm Hg	58.5 (51–66)	52 (44–59)	<0.001
Mean body temperature, °C	36.8 (36.6–37.1)	36.7 (36.4–37)	<0.018
Mean respiratory rate	18.6 (16.4–21.5)	20.8 (17.4–24.5)	<0.001
SPO_2_ min, %	93 (90–95)	90.5 (86–93)	<0.001
Laboratory findings			
Blood glucose max, mg/dL	175 (135.6–244.8)	181 (139.5–277.8)	0.191
Hematocrit min, %	27.9 (23.3–32)	27.5 (23.8–33.6)	0.579
Hemoglobin min, g/dL	9.1 (7.7–10.6)	8.6 (7.5–10.9)	0.607
Platelets min, ×10^3^/μL	171.5 (109.5–248)	158 (70–237.8)	0.037
White blood cell count max, ×10^3^/μL	13.4 (8.9–19.2)	15.7 (8.7–25.8)	0.082
Albumin min, g/dL	3 (2.6–3.5)	2.7 (2.3–3.1)	<0.001
Bicarbonate max, mmol/L	23 (20–26)	21 (18.2–25)	0.007
Blood urea nitrogen max, mg/dL	33 (21–56)	47.5 (31.2–71)	<0.001
Chloride max, mmol/L	105 (102–109)	103 (98–109)	0.007
Creatinine max, mg/dL	1.6 (1–2.9)	2.2 (1.4–3.1)	0.013
Serum sodium max, mmol/L	139 (136–141)	138 (133–142)	0.351
Absolute neutrophils max, ×10^9^/L	10 (5.8–15.6)	11.3 (6.2–17.6)	0.129
Prothrombin time max, s	14.9 (13.1–18.3)	17.5 (14–22.6)	<0.001
Partial thromboplastin time max, s	32.9 (27.8–42.5)	38.5 (30.3–61.5)	<0.001
Aspartate aminotransferase max, U/L	64 (32–168.2)	116 (32.8–480.5)	0.002
Alanine aminotransferase max, U/L	30 (17–73)	55 (23.8–229.2)	<0.001
Bilirubin total max, mg/dL	0.7 (0.4–1.7)	1.1 (0.4–2.9)	0.004
Serum calcium max, mmol/L	8.7 (8.2–9.1)	8.6 (8.1–9.2)	0.719
Creatine kinase max, U/L	209.5 (72–512)	170 (71–867.2)	0.942
Creatine kinase MB Form max, U/L	6 (3–12)	6 (3–12.8)	0.564
Lactate dehydrogenase max, U/L	377.5 (222.8–667)	583.5 (330.5–1287)	<0.001
International normalized ratio	1.4 (1.2–1.7)	1.6 (1.3–2.1)	<0.001
Absolute lymphocytes min, ×10^9^/L	0.9 (0.5–1.4)	0.8 (0.4–1.2)	0.051
Serum potassium max, mmol/L	6.1 (5.7–6.7)	6.2 (5.8–7.0)	0.170
Aniongap max	18 (15–22)	21 (17–25)	<0.001
Urineoutput, mL	1317.5 (800–2427.5)	602.5 (247.8–1093)	<0.001
Lactate max, mmol/L	2.3 (1.6–4)	3.2 (1.7–7.5)	<0.001
pH min	7.3 (7.2–7.4)	7.2 (7.1–7.3)	<0.001
PO_2_ min, mm Hg	53 (36–83)	47 (36.2–70)	0.274
PCO_2_ max, mm Hg	47 (39–53)	49 (42–58.8)	<0.001
Medical treatments			
Ventilation			
No, n (%)	103 (29.4%)	32 (21.9%)	0.109
Yes, n (%)	247 (70.6%)	114 (78.1%)	
Sedative			
No, n (%)	203 (58%)	62 (42.5%)	0.002
Yes, n (%)	147 (42%)	84 (57.5%)	
Vaso			
No, n (%)	229 (65.4%)	59 (40.4%)	<0.001
Yes, n (%)	121 (34.6%)	87 (59.6%)	

The cohort was divided into 2 groups: a mortality group and a non-mortality group. After conducting normality tests on continuous variables, an independent samples *t*-test was used for those adhering to a normal distribution, and the Mann–Whitney *U* test was used for variables that deviated from a normal distribution. Categorical variables were analyzed using the Chi square test or Fisher exact test as appropriate. For statistical descriptions, the study utilized medians and quartiles to compare continuous variables, and frequencies and percentages to compare component variables.

### 3.3. Model development and validation

**Figure 2.**
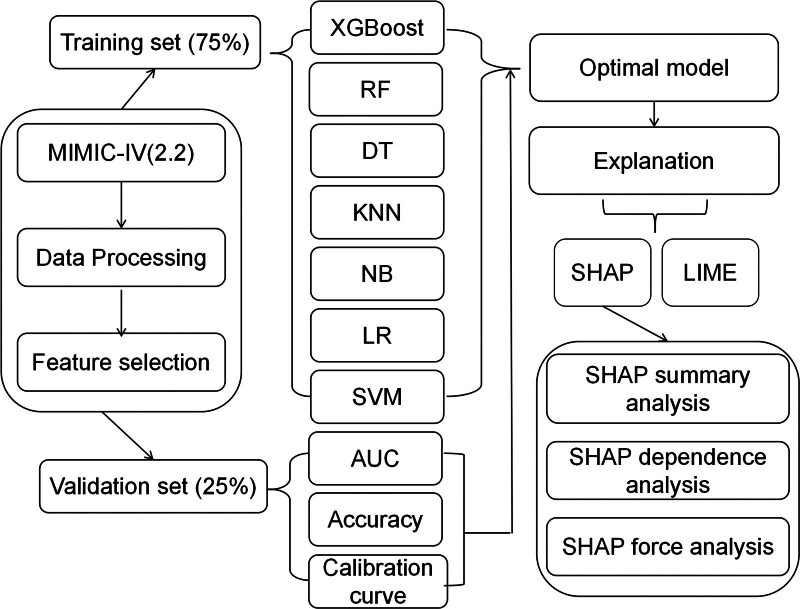
Study procedure flowchart. XGBoost, Extreme Gradient Boosting, RF, Random Forest, SVM, Support Vector Machine, KNN, K-Nearest Neighbors, DT, Decision Tree, NB, Naive Bayes, and LR, Logistic Regression. SHAP, SHapley Additive exPlanations, LIME, Local Interpretable Model-agnostic Explanations.

**Figure 3.**
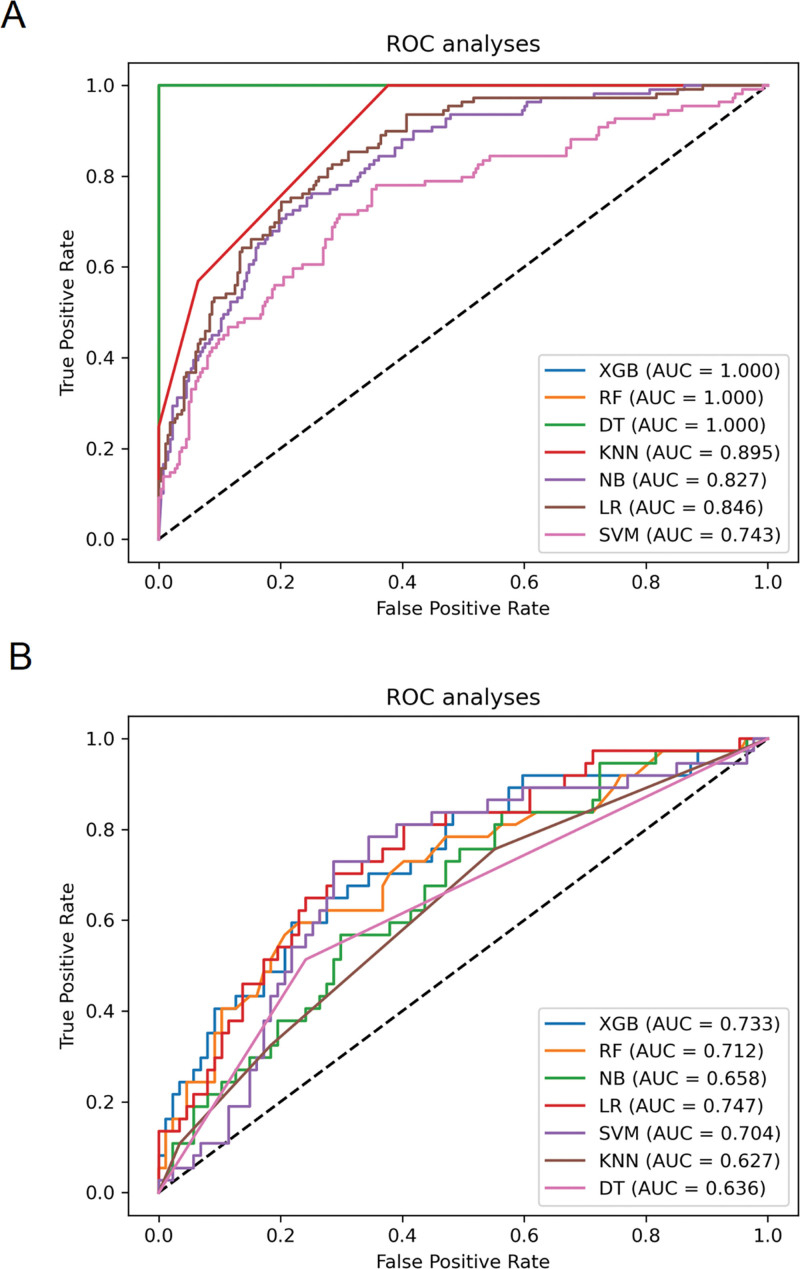
Comparative analysis of machine learning models via ROC Curves: Subfigure A displays the ROC curve results for the training set, while Subfigure B depicts the outcomes for the validation set. XGBoost, Extreme Gradient Boosting, RF, Random Forest, SVM, Support Vector Machine, KNN, K-Nearest Neighbors, DT, Decision Tree, NB, Naive Bayes, and LR, Logistic Regression. AUC, Area Under the Curve, ROC, Receiver Operating Characteristic.

We employed 7 ML algorithms to develop a predictive model for estimating mortality risk in ICU patients with MTH. The flowchart of the study is depicted in Figure 2. The Area Under the Receiver Operating Characteristic Curve (AUC) values of the algorithms are illustrated in Figure 3. We found that XGBoost, RF, and DT got a perfect AUC of 1.000 in the training dataset (as shown in Figure 3A). In the validation dataset, XGBoost AUC was 0.733, which was marginally outperformed by LR AUC of 0.747 (refer to Figure 3B). With respect to accuracy, XGBoost got a score of 1.000 in the training dataset, surpassing LR score of 0.790. The accuracy of all models on the training and validation datasets is detailed in Table 2. Notably, both XGBoost and LR attained 0.734 in the validation dataset, with XGBoost getting 1.000 on the training set, which was higher than LR. Consequently, XGBoost was chosen for the final model development, considering its comprehensive performance. The calibration curves further validated the XGBoost model’s efficacy, evidencing a strong concordance between the prediction and the outcomes (refer to Supplementary Material S3, http://links.lww.com/MD/N90).

**Table 2:** Comparative performance of seven machine learning models across training and validation sets.

ML	Accuracy	AUC
XGboost		
Training set	1.000	1.000
Validation set	0.734	0.733
RF		
Training set	1.000	1.000
Validation set	0.742	0.712
LR		
Training set	0.790	0.846
Validation set	0.734	0.747
DT		
Training set	1.000	1.000
Validation set	0.694	0.636
SVM		
Training set	0.739	0.743
Validation set	0.677	0.704
KNN		
Training set	0.828	0.895
Validation set	0.669	0.627
NB		
Training set	0.774	0.827
Validation set	0.669	0.658

An acronym for every machine learning algorithm: AUC = Area Under the Curve, DT = Decision Tree, KNN = K-Nearest Neighbors, LR = Logistic Regression, NB = Naive Bayes, RF = Random Forest, SVM = Support Vector Machine, XGBoost = eXtreme Gradient Boosting.

### 3.4. Model explanatory analysis

**Figure 4.**
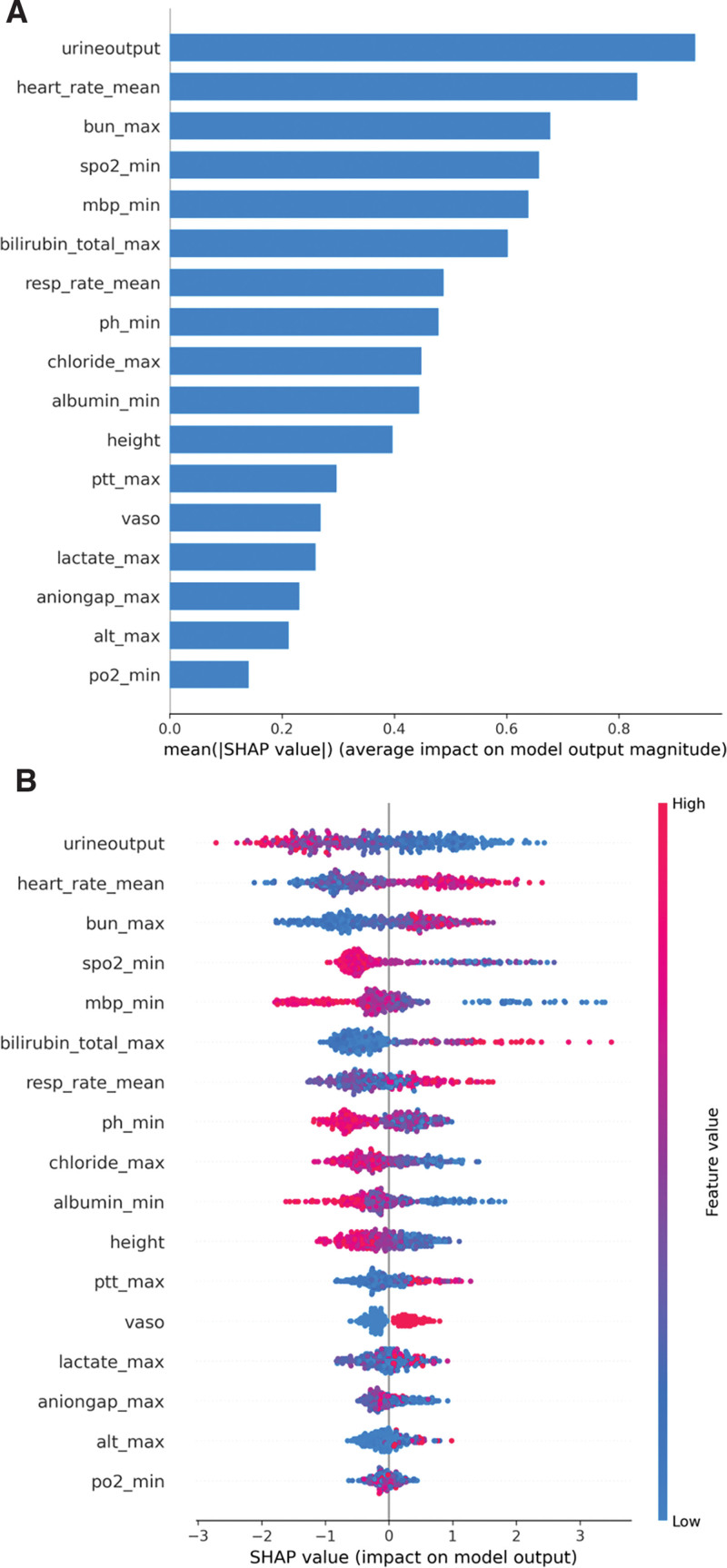
Impact of Top 17 Clinical Predictors in XGBoost Model Assessed by SHAP values: Subfigure A ranks these predictors according to SHAP feature importance, assessed by the mean absolute Shapley values. This heat map illustrates the relative importance of individual predictors within the model. Subfigure B portrays the SHAP value distribution for each predictor, with the *y*-axis enumerating the clinical features and the *x*-axis representing the corresponding Shapley values. SHAP, SHapley Additive exPlanations. urine_output, urine output, heart_rate_mean, mean heart rate, bun_max, maximum blood urea nitrogen, spo2_min, minimum oxygen saturation, mbp_min, minimum mean blood pressure, bilirubin_total_max, maximum total bilirubin, resp_rate_mean, mean respiratory rate, ph_min, minimum pH, chloride_max, maximum chloride, albumin_min, minimum albumin, ptt_max, maximum partial thromboplastin time, vaso, vasopressor use, lactate_max, maximum lactate, aniongap_max, maximum anion, alt_max, maximum alanine aminotransferase, po2_min, minimum partial pressure of oxygen.

In this study, the influence of individual predictors on the predicted outcomes was evaluated by SHAP values generated by the XGBoost model. This model exhibited considerable discriminative capacity within the validation cohort. Figure 4A presents SHAP summary plots for the top 17 clinical features that had the greatest impact on the model’s predictions. These plots illustrate the feature importance based on the mean absolute Shapley values, thereby signifying the relative significance of each variable within the final model. The importance of the 17 clinical features was ranked from highest to lowest for the predictive model as follows: urine output, mean heart rate, maximum blood urea nitrogen, minimum oxygen saturation, minimum mean blood pressure, maximum total bilirubin, mean respiratory rate, minimum pH, maximum chloride, minimum albumin, height, maximum partial thromboplastin time, vasopressor use, maximum lactate, maximum anion gap, maximum alanine aminotransferase, and minimum partial pressure of oxygen. The detailed SHAP summary plots in Figure 4B reveal the distribution of SHAP values for each predictor across all samples, which elucidates the general trends and facilitates the identification of outliers that deviate from predictive norms. Each feature is aligned on the *y*-axis, with its corresponding Shapley value extending along the *x*-axis. The intensity of the color represents the magnitude of a feature’s value, ranging from low (light) to high (dark). The plots distinctly showcase how different covariates positively or negatively influence the model’s predictions. A darker hue signifies a higher actual value of the feature, and its position relative to the 0-axis on the plot indicates the direction of the correlation: leftward for an inverse relationship and rightward for a positive association with the outcome of interest.

**Figure 5.**
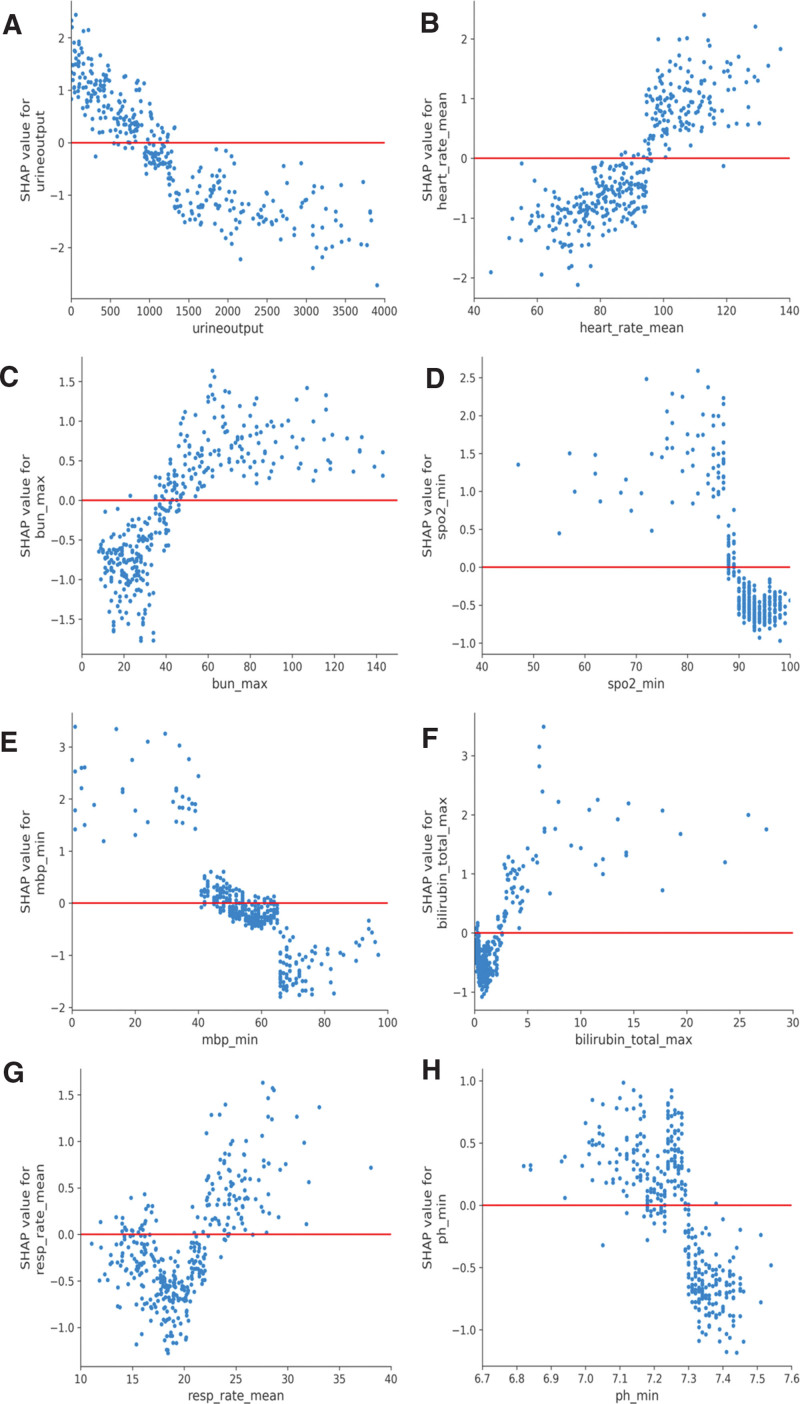
Relationship between clinical indicators and hospital mortality risk. SHAP, SHapley Additive exPlanations. urine_output, urine output, heart_rate_mean, mean heart rate, bun_max, maximum blood urea nitrogen, spo2_min, minimum oxygen saturation, mbp_min, minimum mean blood pressure, bilirubin_total_max, maximum total bilirubin, resp_rate_mean, mean respiratory rate, ph_min, minimum pH.

Through a meticulous examination of the 8 most pivotal predictors using SHAP dependence plots (Fig. 5), we discerned a direct positive correlation between the average heart rate and maximum blood urea nitrogen with the likelihood of mortality. Conversely, urine output, oxygen saturation, and blood pH level exhibited a negative correlation with mortality risk. Minimum mean blood pressure, average respiratory rate, and bilirubin levels each demonstrated a “U” shaped association, indicating a complex relationship with mortality risk wherein both very low and very high values are linked to increased risks.

### 3.5. Examples of model applications

**Figure 6.**
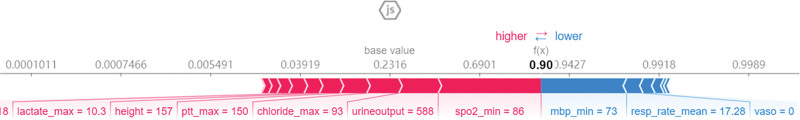
SHAP Force Plot for In-Hospital Mortality Risk Assessment. Factors that increase the risk are highlighted in red, while those reducing or having no effect on the risk are shown in blue. The length of each line segment indicated the magnitude of that factor’s impact. SHAP, SHapley Additive exPlanations. urine_output, urine output, spo2_min, minimum oxygen saturation, mbp_min, minimum mean blood pressure, resp_rate_mean, mean respiratory rate, ptt_max, maximum partial thromboplastin time, vaso, vasopressor use, lactate_max, maximum lactate.

**Figure 7.**
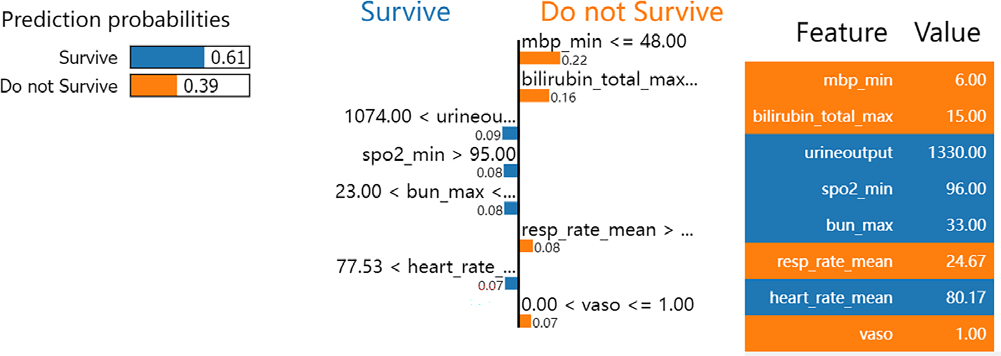
LIME analysis of machine learning model predictions. Orange represents factors that increase the patient’s risk, whereas blue signifies those that reduce or do not affect the risk. LIME, Local Interpretable Model-agnostic Explanations. urine_output, urine output, heart_rate_mean, mean heart rate, bun_max, maximum blood urea nitrogen, spo2_min, minimum oxygen saturation, mbp_min, minimum mean blood pressure, bilirubin_total_max, maximum total bilirubin, resp_rate_mean, mean respiratory rate, vaso, vasopressor use.

A case study involving a randomly selected patient from the validation set was undertaken to highlight the contributions of specific features to individual patient prognoses and to assess the clinical utility of the XGBoost model. The model predicted a 90% mortality risk for this patient, identifying the primary risk factors as minimum oxygen saturation of 86%, a urine output of 588 ml, a peak chloride level of 93 mmol/L, and an elevated partial thromboplastin time of 150 seconds (as shown in Figure 6). Additionally, the LIME method was employed to provide interpretability for the model’s predictions. An analysis of another randomly chosen patient revealed a 39% mortality risk, with critical factors being a minimum mean blood pressure of 6 mm Hg, a maximum total bilirubin of 15 mg/dL, and an average respiratory rate of 24.67 breaths per minute. The use of vasopressors was also noted, all of which collectively increased the patient’s risk of mortality (as depicted in Figure 7).

## 4. Discussion

The global cancer burden is growing; between 2010 and 2019, there was a 26.3% rise in cancer incidence, a 20.9% increase in mortality, and a 16.0% uptick in Disability-Adjusted Life Years (DALYs). The Global Burden of Disease study has positioned cancer as the second leading cause of death, loss of life years, and DALYs worldwide.^[29]^ In the ICU, patients with MTH often exhibit shock, systemic dysregulation, and severe infections, precipitating multi-organ dysfunction syndrome and mortality.^[30]^ Traditional clinical monitoring approaches frequently fail to capture the progression of critical illness and organ function status, and significant pathological damage often occurs before clinical intervention, underscoring the necessity for precise monitoring indicators. Robust ML predictive models have the potential to delineate prognostic risk factors, pinpoint patients at heightened mortality risks, forecast death rates, and inform early, effective therapeutic interventions to mitigate prognostic risk.

This study selected 496 patients with MTH to develop predictive models leveraging 7 ML algorithms. Among these, the XGBoost model was notable for its superior discriminative ability, accuracy, and interpretability, suggesting its utility in aiding clinical decision-making. For the predictive model, 45 clinical and laboratory variables were initially considered, from which LASSO regression identified 17 significant predictors. Feature importance analysis of the XGBoost model highlighted 8 variables as most predictive: urine output, average heart rate, peak blood urea nitrogen, lowest oxygen saturation, lowest mean blood pressure, highest total bilirubin, average respiratory rate, and lowest pH.

Our study findings indicate that in ICU patients with MTH, urine output is a critical determinant of mortality risk. A direct correlation exists between increased mortality risk and reduced urine output, with the risk becoming pronounced when the output falls below 1500 mL/d. Similarly, Shen et al^[31]^ have demonstrated that a severe negative fluid balance, characterized by reduced fluid intake and urine output, is linked to heightened hospital mortality. Hyperkalemia, often resulting from abnormal kidney function or massive tumor cell lysis, is marked by decreased urine output, which thus serves as an indicator of both blood potassium levels and the degree of tumor malignancy. Despite its predictive value, urine output is frequently overlooked in clinical settings. Therefore, clinicians should regularly monitor urine output when managing patients with MTH and consider it an important indicator for assessing patient conditions and adjusting treatment plans. Results showed that a higher average heart rate correlates with increased MTH patient mortality, particularly when it exceeds 100 beats per minute. Consistent with our findings, Pattison’s^[32]^ research associates elevated potassium levels, rapid heart rates, and declining blood oxygen saturation with heightened short-term mortality risk in critically ill cancer patients. Our prediction model finds that blood urea nitrogen levels exhibit a significant positive association with mortality. Blood urea nitrogen serves as an essential biomarker for renal function. This finding is supported by an observational study, which found that patients with advanced neoplasms manifest elevations in blood urea nitrogen levels, a finding consistent with the catabolic state of cachexia.^[33]^ Blood urea nitrogen activates sympathetic and RASS system regulatory mechanisms and regulates renal perfusion and antidiuretic hormone, which induces heart failure and increases the risk of death.^[34,35]^ In this study, we find that the risk of death in ICU patients with MTH reaches its minimum at approximately 18 respiratory rate/minute, and respiratory rate shows a U-shaped relationship with the risk of death. A retrospective cohort study shows that patients with a respiratory rate coefficient of variation ≤ 20% in the first 24 hour during admission to the ICU have a higher risk of ICU and hospital death than those with a respiratory rate coefficient of variation > 20%, and the correlation between low respiratory variability and adverse outcomes may be related to a reduced ability to maintain a stable internal environment following a stress response, and that a high respiratory variability may be a response of the organism to a condition used to maintain stability of the internal environment during stress.^[36]^ The relationship between minimum blood pressure and mortality is U-shaped, with hypotension being a strong prognostic marker for adverse outcomes. When hypotension co-occurs with malignant tumors, the mortality rate further increases.^[37]^ Patients with hyperkalemia using angiotensin receptor–neprilysin inhibitors may also experience hypotension and vasogenic edema, thereby increasing mortality risk.^[38]^ However, the mortality rate increases slightly when the minimal average blood pressure exceeds a certain threshold. Our findings are corroborated by Ettehad et al,^[39]^ whose study shows that high blood pressure is associated with an increased incidence of cardiovascular diseases such as coronary artery disease, stroke, and heart failure, leading to an increased risk of death. Although high bilirubin has long been seen as an indicator of liver dysfunction, recent studies have confirmed that serum bilirubin has potential protective effects against various diseases, including malignant tumors, through antioxidative actions, inhibiting tumor cell proliferation and metastasis and inducing tumor cell apoptosis.^[40]^ According to studies by Inoguchi et al,^[41]^ bilirubin levels relate to mortality in patients with malignant tumors in a U-shaped curve, with the lowest mortality occurring at total bilirubin concentrations around 1.2mg/dL (normal range is 0.1–1mg/dL), which aligns with our findings. However, excessively high bilirubin levels can induce tumor cell apoptosis, releasing intracellular contents that can exacerbate hyperkalemia in patients, which is disadvantageous for patient survival.^[42]^ Erdur et al^[43]^ find that in ICU patients with malignant tumors, the pH value in the deceased group is significantly lower than that in the survivors. Schork et al^[44]^ discover that the median of the minimum pH value within 24 hours in the deceased group is 7.28, significantly lower than 7.38 in the non-deceased group. Our study also indicates that when the minimum blood pH value falls below 7.3, the patient’s risk of death rises sharply. This is broadly consistent with the findings of the 2 studies. Cancer cells’ aberrant energy metabolism, abnormal proliferation, or insufficient perfusion can create significant extracellular acidity, which promotes cancer cell migration. A low pH value can also inhibit immune cell infiltration, potentially fostering cancer progression. Thus, maintaining an appropriate pH value is critically important for the prognosis of ICU patients with malignant tumors and concurrent hyperkalemia.^[45]^

Our study highlights the importance of precise monitoring and managing physiological parameters in patients with MTH. By integrating robust predictive models into clinical practice, healthcare providers can improve prognostic assessments and tailor interventions more effectively to reduce mortality risks. However, we acknowledge potential biases and confounding factors that could have influenced our results. The retrospective nature of this study and the specific ICU setting may limit the generalizability of our findings. Insufficient sample size may also lead to weak generalization and reduced accuracy, affecting the model’s effectiveness in practical applications. Future studies should adopt prospective designs and incorporate a variety of clinical settings to validate and enhance the predictive accuracy of the identified markers. Additionally, it is imperative to involve a larger sample size to validate and refine the model, ensuring its accuracy and generalizability. Exploring the integration of these models with real-time monitoring systems in ICUs could enable dynamic updates to risk assessments and treatment plans, potentially leading to more personalized patient care. Further studies are also suggested to assess the effectiveness of these models in improving clinical outcomes and explore integrating these models into clinical decision support systems to enhance decision-making quality and reduce the incidence of adverse events.

## 5. Conclusion

In this study, we employed the XGBoost ML algorithm for the first time to develop and validate a predictive model for predicting risks of in-hospital mortality among ICU patients with MTH, incorporating a total of 17 predictors. By utilizing interpretative methods such as SHAP and LIME, we enhanced the explainability of our model. Our study highlights the critical role of predictors such as urine output and mean heart rate in determining the risk of death in patients with MTH. This finding suggests that clinicians should pay special attention to the dynamics of these predictors in MTH patients. These monitoring measures are essential for the active clinical management of MTH patients and can prompt physicians to intervene in a timely manner, which may significantly improve patient outcomes. However, it must be noted that although our model has demonstrated initial predictive capabilities, future studies are suggested to involve larger sample sizes to further validate and refine the model to ensure its accuracy and generalizability.

## Acknowledgments

We are very grateful to instructors Zhao-Lan Liu and Jian-Ping Liu of the Centre for Evidence-Based Medicine, Beijing University of Chinese Medicine, for their help with our manuscript. We extend our gratitude to all authors for their contributions to this study and thank them for their diligent efforts.

## Author contributions

**Conceptualization:** Zhi-Jun Bu.

**Data curation:** Zhi-Jun Bu, Nan Jiang, Ke-Cheng Li, Zhi-Lin Lu, Zu-Yi Chen.

**Formal analysis:** Zhi-Jun Bu, Nan Jiang, Ke-Cheng Li, Zhi-Lin Lu.

**Methodology:** Zhi-Jun Bu, Nan Jiang, Ke-Cheng Li, Zhi-Lin Lu, Shao-Shuai Yan, Zhi-Lin Chen, Yu-Han Hao, Yu-Huan Zhang, Run-Bing Xu, Han-Wei Chi.

**Project administration:** Zhi-Jun Bu, Jian-Ping Liu, Dan Wang, Feng Xu, Zhao-Lan Liu.

**Software:** Zhi-Jun Bu, Nan Jiang, Ke-Cheng Li, Zhi-Lin Lu, Shao-Shuai Yan, Zhi-Lin Chen, Yu-Han Hao, Yu-Huan Zhang, Run-Bing Xu, Han-Wei Chi.

**Validation:** Zhi-Jun Bu, Nan Jiang, Ke-Cheng Li, Zhi-Lin Lu, Zu-Yi Chen.

**Visualization:** Zhi-Jun Bu.

**Writing – original draft:** Zhi-Jun Bu, Nan Zhang.

**Investigation:** Nan Jiang, Ke-Cheng Li, Zhi-Lin Lu, Shao-Shuai Yan, Zhi-Lin Chen, Yu-Han Hao, Yu-Huan Zhang, Run-Bing Xu, Han-Wei Chi.

**Resources:** Nan Jiang, Ke-Cheng Li, Zhi-Lin Lu, Shao-Shuai Yan, Zhi-Lin Chen, Yu-Han Hao, Yu-Huan Zhang, Run-Bing Xu, Han-Wei Chi.

**Writing – review & editing:** Nan Zhang.

**Funding acquisition:** Jian-Ping Liu, Dan Wang, Feng Xu, Zhao-Lan Liu.

**Supervision:** Jian-Ping Liu, Dan Wang, Feng Xu, Zhao-Lan Liu.

## Supplementary Material
