# Supplementary material for: Development and Validation of an Interpretable Machine Learning Model for Early Prognosis Prediction in ICU Patients with Malignant Tumors and Hyperkalemia
Source: Medicine (Baltimore). 2024 Jul 26;103(30):e38747. doi: 10.1097/MD.0000000000038747 (PMC11272258; doi:10.1097/MD.0000000000038747)
Supplement: Supplementary file 1 [file medi-103-e38747-s001.docx]

**Supplementary material S1**

**A**

**
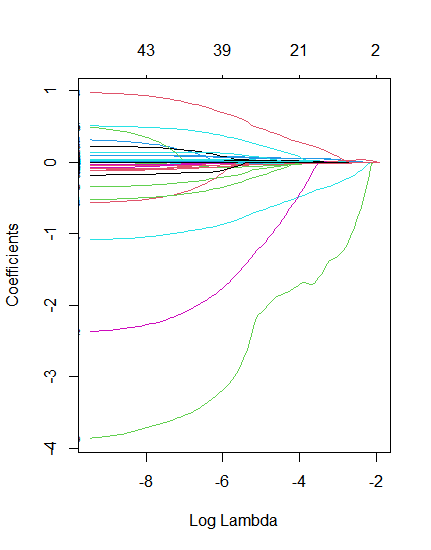
**

**B**

**
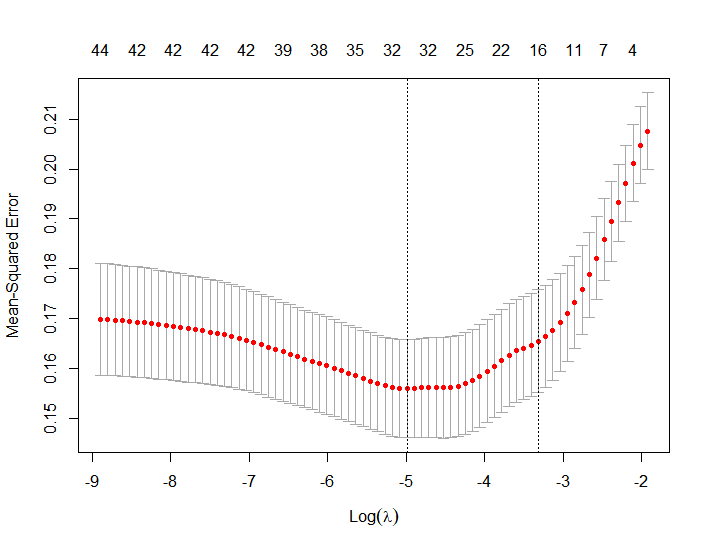
**

Figure S1: Visualization of Feature Selection via LASSO Regression. Panel A: Coefficient Paths illustrates how the coefficients of the predictors change with varying levels of the tuning parameter. The x-axis denotes the tuning parameter values, while the y-axis corresponds to the magnitude of the predictors' coefficients. Panel B: Tuning Parameter vs. Validation Error demonstrates the error associated with each value of the tuning parameter during LASSO regression. Here, the x-axis labels the tuning parameter values, and the y-axis reflects the cross-validation error magnitude.
