# Supplementary material for: Development and Validation of an Interpretable Machine Learning Model for Early Prognosis Prediction in ICU Patients with Malignant Tumors and Hyperkalemia
Source: Medicine (Baltimore). 2024 Jul 26;103(30):e38747. doi: 10.1097/MD.0000000000038747 (PMC11272258; doi:10.1097/MD.0000000000038747)
Supplement: Supplementary file 2 [file medi-103-e38747-s002.docx]

**Supplementary material S2**

**
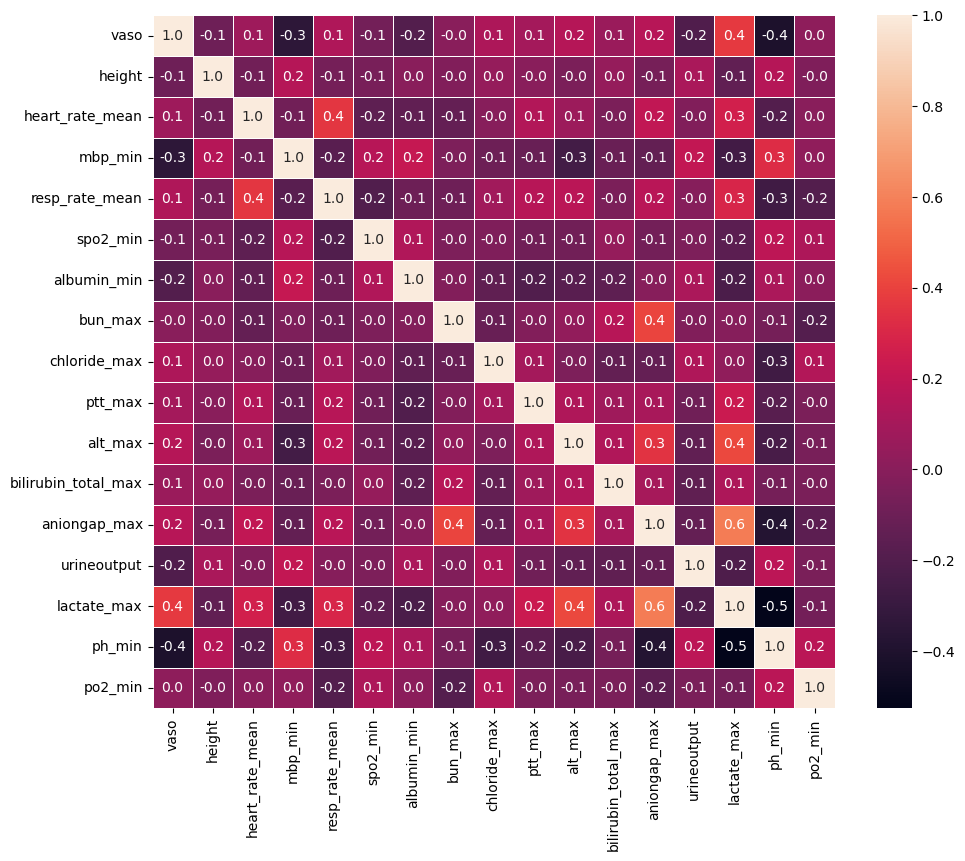
**

Figure S2: The heatmap, created with Python's Seaborn library, reflects Pearson's correlation coefficient for statistical backing. Intensified shades of red signify greater correlation among the features.
