# Supplementary material for: Development and Validation of an Interpretable Machine Learning Model for Early Prognosis Prediction in ICU Patients with Malignant Tumors and Hyperkalemia
Source: Medicine (Baltimore). 2024 Jul 26;103(30):e38747. doi: 10.1097/MD.0000000000038747 (PMC11272258; doi:10.1097/MD.0000000000038747)
Supplement: Supplementary file 3 [file medi-103-e38747-s003.docx]

**Supplementary material S3**

**
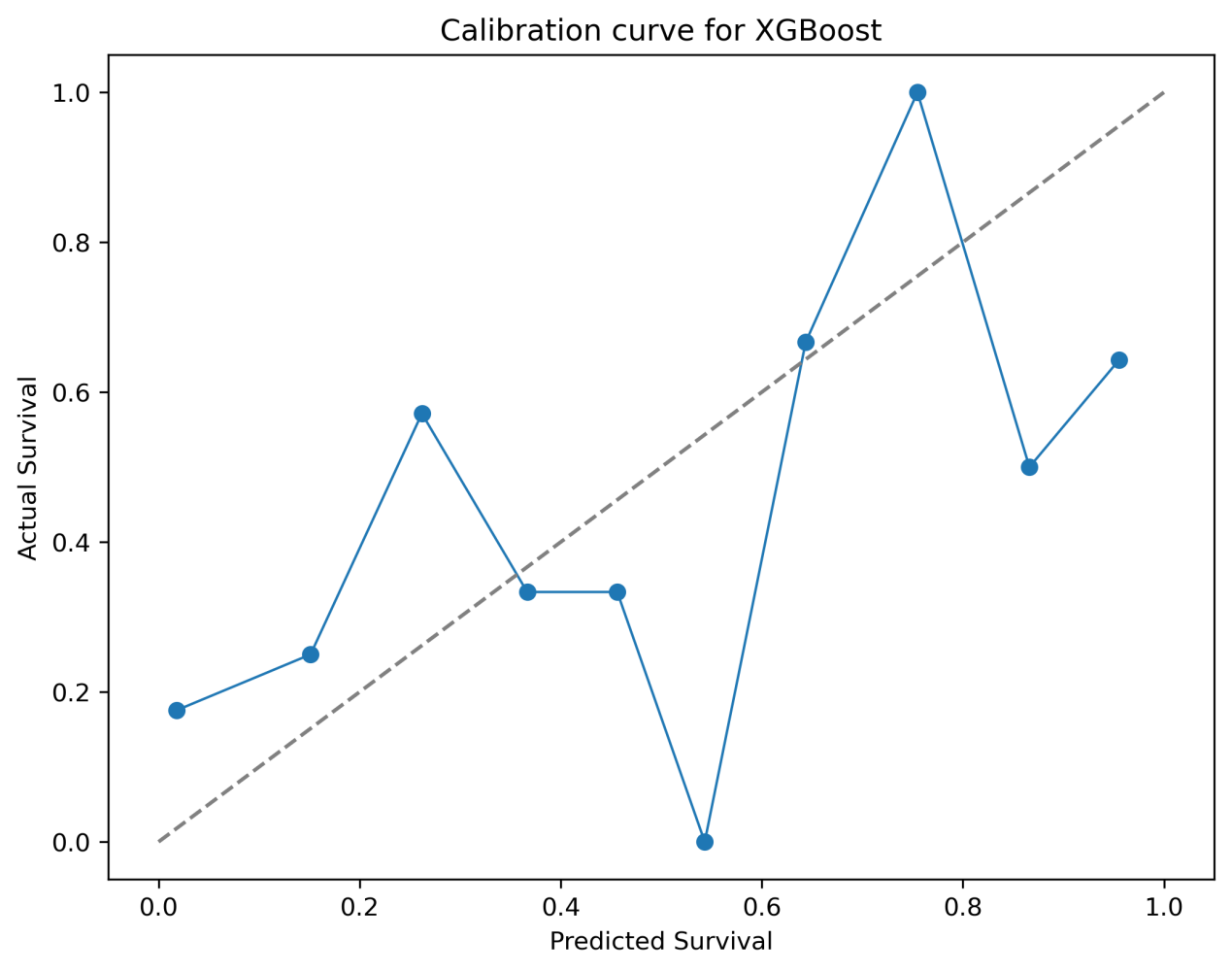
**

Figure S3: This figure presents the calibration plot for the XGBoost model applied to the validation data. It conveys how the model's predicted probabilities align with the observed realities, serving as a gauge for the model's calibration quality. Optimal calibration is indicated by the curve's proximity to the dashed diagonal reference line.
